# Redox Hyperactive MOF for Li^+^, Na^+^ and Mg^2+^ Storage

**DOI:** 10.3390/molecules27030586

**Published:** 2022-01-18

**Authors:** Hristo Rasheev, Agnieszka Seremak, Radostina Stoyanova, Alia Tadjer

**Affiliations:** 1 Faculty of Chemistry and Pharmacy, University of Sofia, 1164 Sofia, Bulgaria; seremak.aga@gmail.com (A.S.); tadjer@chem.uni-sofia.bg (A.T.); 2 Institute of General and Inorganic Chemistry, Bulgarian Academy of Sciences, 1113 Sofia, Bulgaria; radstoy@svr.igic.bas.bg; 3 Faculty of Chemistry, Wroclaw University of Science and Technology, 50-373 Wroclaw, Poland

**Keywords:** Ni(II) node, 2,5-dicyano-*p*-benzoquinone, rechargeable metal-ion batteries, lithium, sodium, magnesium, periodic DFT calculations, electrode potential

## Abstract

To create both greener and high-power metal-ion batteries, it is of prime importance to invent an unprecedented electrode material that will be able to store a colossal amount of charge carriers by a redox mechanism. Employing periodic DFT calculations, we modeled a new metal-organic framework, which displays energy density exceeding that of conventional inorganic and organic electrodes, such as Li- and Na-rich oxides and anthraquinones. The designed MOF has a rhombohedral unit cell in which an Ni(II) node is coordinated by 2,5-dicyano-*p*-benzoquinone linkers in such a way that all components participate in the redox reaction upon lithiation, sodiation and magnesiation. The spatial and electronic changes occurring in the MOF after the interaction with Li, Na and Mg are discussed on the basis of calculated electrode potentials versus Li^0^/Li^+^, Na^0^/Na^+^ and Mg^0^/Mg^2+^, respectively. In addition, the specific capacities and energy densities are calculated and used as a measure for the electrode applicability of the designed material. Although the highest capacity and energy density are predicted for Li storage, the greater structural robustness toward Na and Mg uptake suggests a higher cycling stability in addition to lower cost. The theoretical results indicate that the MOF is a promising choice for a green electrode material (with <10% heavy metal content) and is well worth experimental testing.

## 1. Introduction

Matching the high-power and large-scale energy storage with environmental constraints entails to consider replacement of the current lithium-ion batteries (LIBs) with technologies, in which more abundant, cheaper and safer elements are used [1,2,3,4]. In this respect, sodium- and magnesium-ion batteries (SIBs and MIBs) are the most prospective alternatives [5,6]. Both SIBs and MIBs operate by the same mechanism as LIBs, comprising the reversible exchange of Na^+^ and Mg^2+^ ions between two electrodes via electrolyte [7,8]. Given that sodium and magnesium have higher redox potentials versus SHE than lithium (−2.71 V for Na^0^/Na^+^ and −2.37 V for Mg^0^/Mg^2+^ compared to −3.04 V for Li^0^/Li^+^ [9]), the enhancement of the energy density of SIBs and MIBs can only be achieved through the advancement of a new class of electrode materials that have the unique property of colossal specific capacity (i.e., able to exchange a huge amount of Na^+^ or Mg^2+^ ions)—a phenomenon not feasible for conventional electrodes based on transition metal oxides and phosphates. This particularity follows from the ability of the electrode material to provide simultaneously flexible structural sites for storage and diffusion of large amounts of Na^+^ or Mg^2+^ ions, as well as to compensate through high redox activity the charges of all transportable metal ions.

Metal-organic frameworks (MOFs) are a specific class of materials that could meet the above requirements due to their structural and redox versatility [10,11,12,13,14]. In contrast to purely organic battery materials [15,16,17], through the bonding of metal-containing nodes with polyfunctional organic linkers, MOFs are prone to form highly ordered structures displaying an enormous variety of arrangements, providing a diversity of diffusion paths for both alkali and alkaline earth ions [18,19,20,21]. The redox activity of MOFs can be differentiated depending on whether the metal centers or the organic ligands or both the metal and the organic entities are active [22,23,24,25]. For MOFs with redox-active metal centers (such as Fe^2+^/Fe^3+^, Cu^+^/Cu^2+^, V^4+^/V^5+^, Co^2+^/Co^3+^, etc.), the number of inserted ions (e.g., alkali or alkaline earth ions) is limited up to 1 mole per metal center [26,27]. This type of redox reaction mimics the operation of the conventional inorganic electrode materials. When the organic ligands take part in the redox reaction, more transportable ions can be inserted, and higher potentials can be achieved [23]. Further advantages of the redox activity of the organic ligands are associated with restricted bond rearrangement during metal ion insertion resulting in small structural changes in comparison with inorganic oxide and phosphate electrodes. Moreover, the participation of the organic ligands in the redox reaction is less dependent on the radius and charge of alkali or alkaline earth ions than in the case of inorganic electrodes.

The most suitable organic ligands included in redox reactions are those containing one or more electron-withdrawing groups like carbonyl and nitrile ones [28]. Of particular interest are the quinones—a class of carbonyl compounds derived from aromatic hydrocarbons. Both theoretical and experimental investigations have shown that quinone-based structures have excellent qualities for application in batteries with high stability/energy density/capacity [29,30,31,32,33,34,35,36,37]. Thus, a viable strategy to build a MOF for energy storage application is to use quinone derivatives functionalized with coordinating groups as organic linkers in combination with suitable metal ions. In order to increase the energy density of the material, the metal ions, if appropriately selected, can also participate in the redox reactions. This has been shown to work in a practical cell comprising Cu^+^/Cu^2+^ redox couple and two different organic linkers—anthraquinone dicarboxylate [23] and tricarboxyphenylamine [38], the latter achieving the very high potential of 4.3 V. Furthermore, Gu et al. [39] applied the previously synthesized conductive MOF—Cu_3_(2,3,6,7,10,11-hexahydroxytriphenylene)_2_ [40] as cathode material. The combination of Li-ion insertion capability with electronic conductivity resulted in an outstanding rate performance. A Cu(II)-based 2D MOF was shown to be able to accommodate lithium as well, while demonstrating high conductivity in both pristine and lithiated forms [41]. Other examples of a redox-active metal-ion couples are V^4+^/V^5+^ in a layered oxalatophosphate [42] and Fe^2+^/Fe^3+^ in a 1,4-benzenedicarboxylate-based MOF [26] for Li-ion batteries; Fe^2+^/Fe^3+^ [27,43] and Co^2+^/Co^3+^ [27] in Prussian-blue analogues for Na-ion batteries. Moreover, new MOF-based electrode materials for anodes in LIB (with low potential vs. Li^0^/Li^+^) have been synthesized and tested. Li et al. [44] reported a Zn/1,3,5-benzenetribenzoate-based MOF but electrochemical testing of the material resulted in a large irreversible capacity loss after the first cycle. The 2,6-naphthalene(COOLi)_2_ intercalation MOF has proven to have better cycling performance (96% capacity retention after 100 cycles) and low potential of 0.8 V vs. Li-metal [45].

Although nickel metal is widely used in battery technologies, its integration into redox-active MOFs is rare. Until now, there have been few reports, including the paper of Hameed et.al. [46], in which a Ni-based 1D-MOF/reduced graphene oxide composite was tested as electrode material in LIB. Nickel(II)-terephthalate coordination complexes have been shown to participate in a multi-electron redox process due to the change in the oxidation state of Ni and interaction of additional Li with the conjugated aromatic ligands [47].

Given the enormous variety of conceivable MOF structures, the exploration of their application as components of metal-ion batteries is still in its infancy. Here, we present a theoretical model of a MOF that is able to store both alkali and alkaline earth metals. Looking for small and light building elements, we have suggested an unreported (to our knowledge) MOF. The building elements are 2,5-dicyano-*p*-benzoquinone (2CN-BQ) used as a redox-active linker and Ni(II) serving as a metal node. The advantage of the proposed MOF over other MOF structures has three aspects. First, we have selected *p*-benzoquinone instead of *o*-benzoquinone in order to achieve consistent output voltages [31]. Then, the *p*-benzoquinone functionalization with CN-groups increases the overall electrochemical potential for metal ion storage together with the opportunity for monodentate coordination with Ni(II), allowing a 3D architecture of the MOF. Finally, the designed MOF is tested for its ability to store Li^+^, Na^+^ and Mg^2+^—a combination that has not been reported so far. The structural characterization of the MOF and its affinity towards Li, Na and Mg are quantified by DFT periodic calculations. The electrode potentials versus Li^0^/Li^+^, Na^0^/Na^+^ and Mg^0^/Mg^2+^, as well as the specific capacities and energy densities, are evaluated and used as a measure for the electrode applicability of the designed material. It has been shown that the suggested MOF can accommodate a huge number of Li and Na atoms (and a more modest number of Mg) with a remarkable charge transfer, exhibiting very promising capacities and energy densities with a great potential for electrode application.

## 2. Results and Discussion

### 2.1. Structure of Pristine MOF-S21

**Figure 1:**
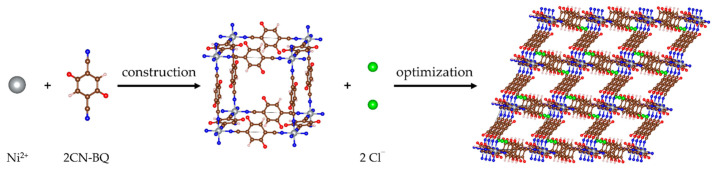
Construction of the unit cell of MOF-S21 and 3D (3 × 3 × 3) multiple cell representation of the optimized MOF. Color code: brown—C; ecru—H; red—O; blue—N; green—Cl; grey—Ni.

The unit cell of the pristine 3D-MOF model was constructed as a cubic cell with an octahedral Ni(II) at the vertices and 2,5-dicyano-*p*-benzoquinone (2CN-BQ) molecules as edges along the three lattice axes (Figure 1). The structure was neutralized by two Cl^−^ as counterions. Periodic boundary conditions were applied. After geometry optimization, a rhombohedral structure ($R\bar{3}$) was obtained with unit cell parameters a = 11.67 Å and α = 56.16°, and volume 1024.8 Å^3^; such distortion has been witnessed experimentally in other coordination polymers of Ni(II) with linear bifunctional ligands (Au(CN)_2_^−^) [48]. The nickel ion retained an octahedral coordination with the nitrogens from the cyano-groups (Figure 1). Because of the trigonal distortion, the initial pore volume underwent a strong shrinkage (i.e., of around ¼). This is an indication for unfeasibility of interpenetration pathways for the designed MOF (Figure 1). Although the pore sizes are not suitable for interpenetration, there are still a plethora of sites for metal ion insertion. Further on, the 3D-structure will be denoted as MOF-S21.

The constructed MOF was computationally tested as an electrode via successive introduction of three different metal atoms—lithium, sodium and magnesium. Important characteristics that can be estimated by means of molecular modelling are the capacity of the material toward the tested metals, the electrode potential and the charge distribution in the system, depending on the degree of metal uptake.

### 2.2. Metal Loading into MOF-S21

The computational procedure to test the prospect of MOF-S21 to serve as electrode material has the following main stages: (i) finding the most favorable structure for every degree of metal insertion; (ii) calculating the internal energy change between two consecutive degrees of metal insertion according to Equation (1); (iii) estimating the electrode potential against the respective metal according to Equation (2).

#### 2.2.1. Lithiation of MOF-S21

**Figure 2:**
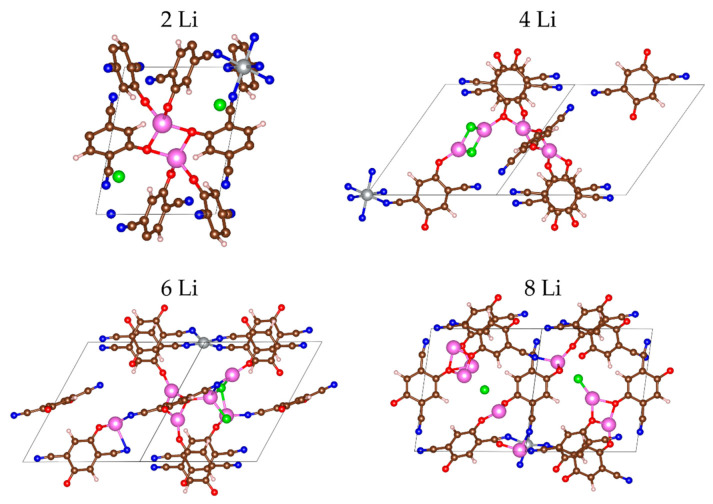
The MOF-S21 unit cell early stages of lithiation. Color code pink—Li, the remaining colors as in Figure 1.

The main redox-active parts of the pristine MOF are the cyano-quinones; they are expected to coordinate Li^+^ via their oxygens, so that in the Li_n_MOF structures, n = 1 through 6, Li-atoms were placed next to the oxygens. For each next degree of metal insertion, several initial structures were optimized; only the results for the lowest-energy ones were considered for further discussion. The optimized lithiated structures are visualized on Figure 2 and Figure S1 (Supplementary Materials). All electro-negative elements (O, N, Cl) participate in the coordination of Li^+^.

In the first step of lithiation (two Li atoms), lithium is coordinated only to oxygens from the C=O groups. In the next step (four Li atoms), the chloride counterions are included in the coordination as well. It is noteworthy that in this step, two Li are coordinated by four oxygens—two individual and two shared for each, while the other two Li share the two chloride ions and coordinate to one individual oxygen (Figure 2). The strive of Li^+^ to be tetracoordinated with four covalently bound oxygens or to be tricoordinated if a halide ion occupies one of the coordination sites has been registered already in an earlier study [49]. Only after the insertion of 6 or more Li atoms all functional groups and counterions are involved in the coordination. The MOF was able to accommodate 24 Li atoms before the energy change became unfavorable.

The inserted Li atoms reduce initially only the organic molecules but upon loading of 6 Li the Ni charge gradually starts to drop (Table 1) and decreases from 1.25 in the empty MOF to 0.24 at 24 Li. For 26 Li atoms the Ni charge becomes close to zero, meaning that it is decoordinated, which is one of the reasons further lithiation is energetically unfavorable.

**Figure 3:**
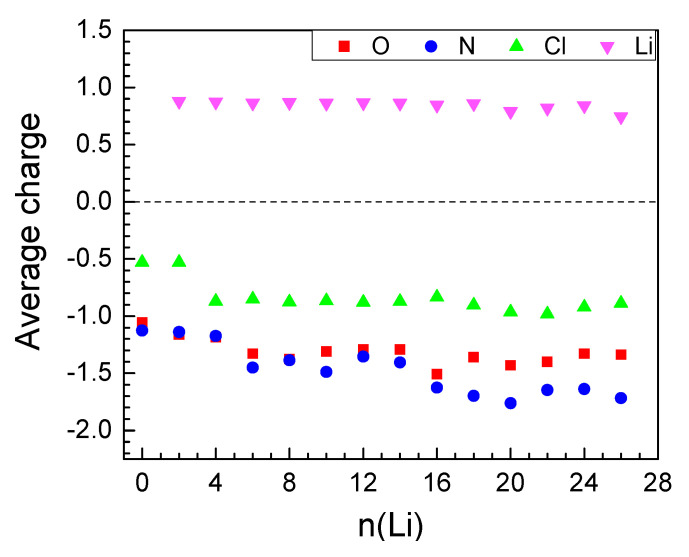
Average AIM charges upon lithiation of MOF-S21.

**Figure 4:**
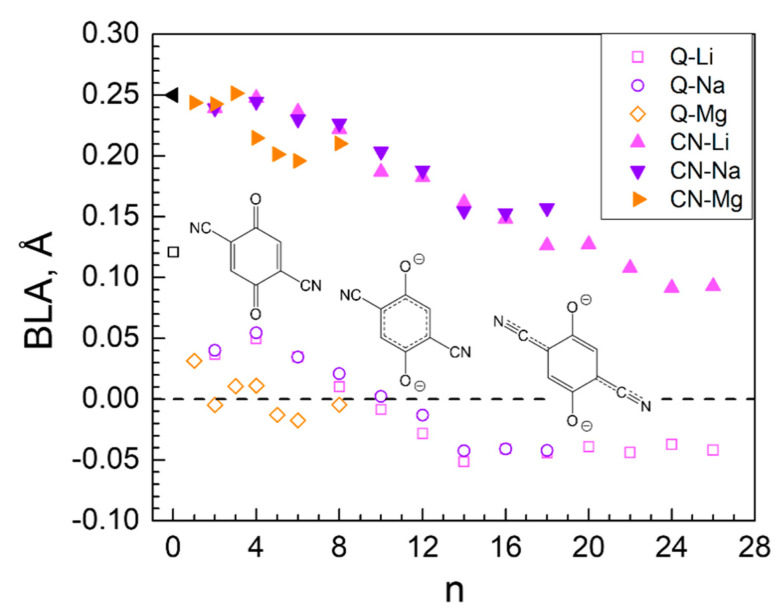
Bond length alternation (BLA) of the C-C bonds in the benzene ring (Q-M) and of the exocyclic C-C≡N bonds (CN-M) upon metal insertion.

Li charge varies in a narrow interval of values throughout the insertion of the first 24 Li atoms and sharply decreases thereafter (Table 1, Figure 3). Electron density is transferred from Li mainly to the electronegative atoms—oxygens and nitrogens (Figure 3), in accordance with the pattern of Li^+^ coordination. Figure 3 shows that O and N bear a similar amount of electron density up to 16 Li and, thereafter, more negative charge is allocated on the nitrogens. The active involvement of nitrogen in charge exchange with Li is the reason for its gradual partial decoordination from the Ni ions—starting at 8 Li, the Ni coordination number drops from 6 to 4 and decreases further down to 2 (Table 1). Parallel to this, close contacts Li-Li (<2.67 Å, the bond length in Li_2_) are registered, growing in number with lithium loading. These non-ionic clusters bear no charge; this is one of the reasons for the gradual decrease of the average Li charge (Table 1, Figure 3 and Figure 4—bottom). This is an interesting result if we refer to the experimentally observed Li clusters that are formed inside the pores of carbon-based anodes (such as hard carbons, microporous carbon with open porosity) upon overlithiation [50,51]. The energy storage by the formation of Li clusters is of practical importance in order to achieve electrode materials with colossal capacity. In this respect, further studies will be needed to understand whether the Li clustering is a specific feature of MOF-S21.

The effect of Li insertion on the periodic box proportions is presented in Table 2. Metal insertion causes lowering of the lattice symmetry due to variation of all lattice parameters. In more detail, the volume of the box shrinks during the insertion of the first 6 Li atoms, more prominently at the expense of angle reduction, then expands again due to angle growth until 16 Li, and finally contracts again due to both edge and angle decrease. Owing to this ‘breathing’ behavior, the volume oscillates (Table 1) but stays lower than the initial one at any degree of Li loading. At the maximum Li uptake, the volume is the same as for 2Li—~20% less than that of the pristine MOF. Irrespective of this dimension variation, the designed MOF is sufficiently stable to accommodate a huge amount of Li.

The changes in the edge lengths were due to the changes in the geometry of the ligands resulting from the charge redistribution in the system. A characteristic feature of the initial structure of the MOF edges is the bond length alternation (BLA), which is defined in principle as the average difference between the longer and the shorter bonds in a π-conjugated molecule. We have considered two values of BLA: (i) the BLA of the C-C bonds in the cyclic portion of the quinones (Q-M) and (ii) the alternation of the lengths of the triple C≡N and the adjacent simple C-C exocyclic bonds (CN-M). The results are shown in Figure 4 and reveal that, upon Li loading, the quinoid ring gradually becomes aromatic, as the BLA reaches zero at 10 Li, and, further on, becomes negative, i.e., the former shorter bonds become longer and vice versa. On the other hand, the BLA of the exocyclic fragment drops drastically, indicating the tendency towards equalization of the two bonds. As a result, the quinoid character of the cycle is restored to a certain degree but in another direction and the overall effect is shortening of the edges. Alongside the change in geometry, the organic ligand changes slightly its orientation due to partial decoordination from Ni and coordination to Li^+^. This results in reduction of the Ni-Ni distances and in overall volume decrease.

**Figure 5:**
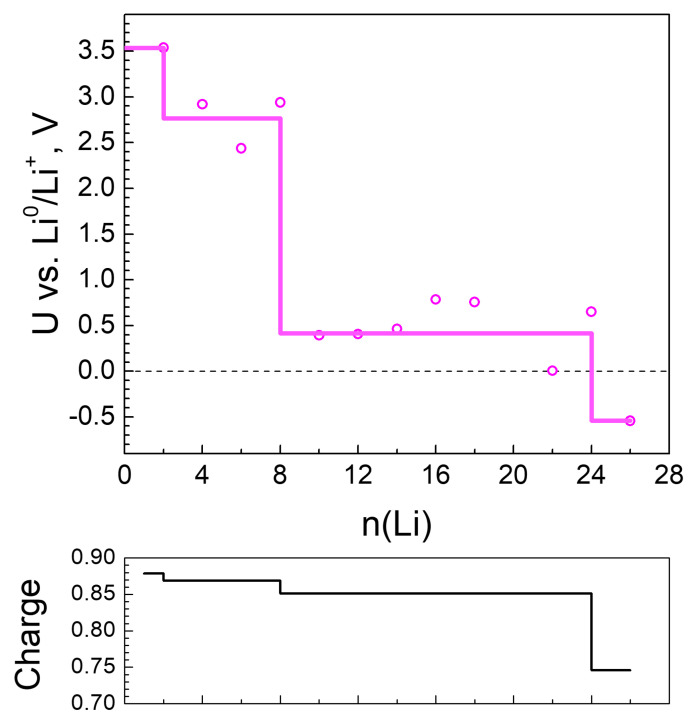
Calculated electrode potential profile (U vs. Li^0^/Li^+^) compared to Li charge variation as a function of lithiation.

Based on the above geometric and electronic analysis, it is now possible to understand the reduction properties of the lithiated MOF-S21. Figure 5 shows the calculated electrochemical potential of the material versus Li^0^/Li^+^. The MOF reduction by Li proceeds at three distinct potential steps: at ~3.5 V interact the first 2 Li atoms, followed by a potential at 2.8 V (4 Li to 8 Li), and finally a stable potential at 0.4 V is developed (beyond 8 to 24 Li). This ’terrace-wise’ change in the electrochemical potential upon lithiation mirrors the Li charge profile (Figure 5-bottom), which, on its turn, reflects the pattern of Li^+^ coordination in the MOF. It is interesting to note that a two-step reduction has been experimentally observed upon the lithiation of 2,3-dicyano-o-benzoquinone (o-2CN-BQ): 3.37 and 2.46 V, respectively [52]. The good agreement between the electrochemical potentials calculated for the lithiated MOF and the measured potential of o-2CN-BQ gives further evidence that the MOF reduction is dominated by the 2CN-BQ structural element during the initial degrees of lithiation. In addition, the MOF accommodates extra Li atoms in the third potential step (i.e., at 0.4 V). This is achieved through the activation of the redox properties of the Ni ion in addition to the CN-nitrogens. At 8 Li the formation of Li clusters starts (Figure 4). It is clear that a combination between the redox properties of Ni and 2CN-BQ, as well as the MOF flexible structure, bear promise for achieving a very high lithium capacity.

#### 2.2.2. Sodiation of MOF-S21

**Figure 6:**
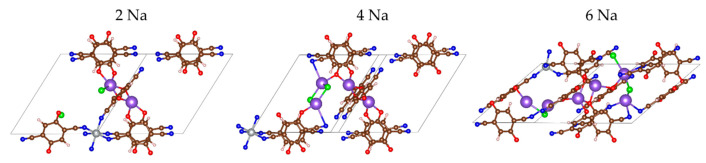
The MOF-S21 unit cell early stages of sodiation. Color code: violet—Na, the remaining colors as in Figure 1.

The initial placement of the Na atoms followed the same logic as Li—next to oxygens. However, the optimized geometries did not coincide ideally—as early as the first pair of Na atoms, the Cl^−^ counterion was already involved in the coordination of Na^+^ and at 4 Na the CN-nitrogens joined in as well (Figure 6, Figure S2 and S3). Sodium has a flexible coordination number but is definitely higher than 4, which explains the coordination to more electron-acceptors.

**Figure 7:**
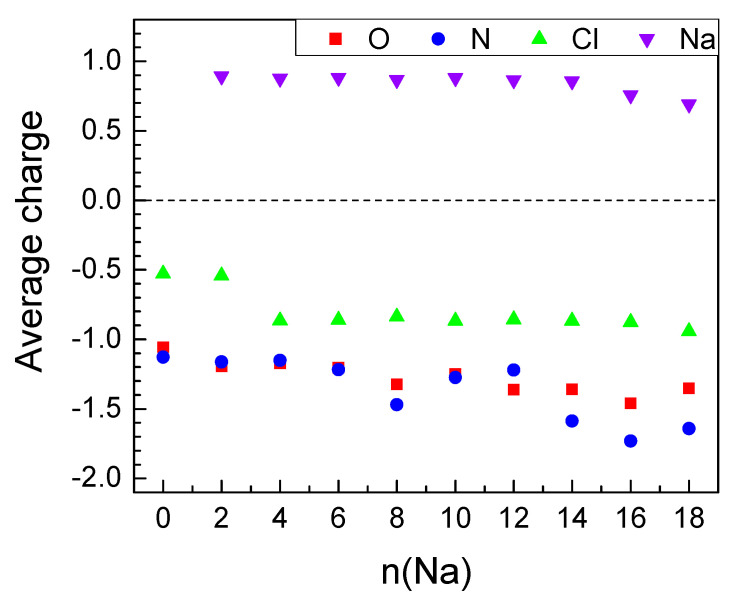
Average AIM charges upon sodiation of MOF-S21.

**Figure 8:**
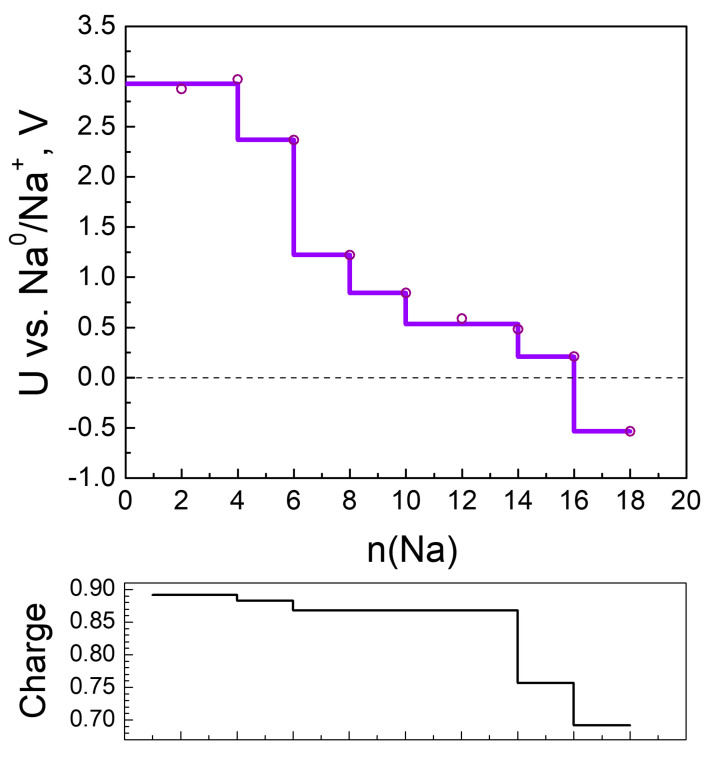
Calculated electrode potential profile (U vs. Na^0^/Na^+^) compared to Na charge variation as a function of sodiation.

The effect of Na insertion on Ni is both similar to and different from Li insertion. The charge of Ni decreases faster until 8Na but keeps almost constant thereafter, even when insertion becomes unfavorable (Table 3). The Na charge maintains a high essentially constant value upon loading of the first 14 Na and only decreases in the last steps (Table 3, Figure 7 and Figure 8—bottom). The electron density is again transferred from Na to oxygen and nitrogen but more uniformly than in the case of Li. Only in the last steps do the nitrogens accept more charge than the oxygens. As a result, the Ni coordination number drops from 6 to 4 and does not decrease further (Table 3). This is the reason why the effects of Ni decoordination on the geometry of the periodic box are different (Table 2) and tend to rectify the structure back to a cubic box. The volume again shrinks during the insertion of the first 6 Na by 17% but then expands and maintains an almost constant value at about 95% of the empty box (Table 3). The BLA values for both Q-Na and CN-Na (Figure 4) have exactly the same conduct as the respective Q-Li and CN-Li—aromatization of the quinoid ring upon insertion of 8–12 Na and inversion of the direction of quinoidization thereafter. However, a major difference from the Li uptake behavior is the absence of clusters—only one close Na-Na contact is found in the last three favorable insertion steps (Table 3).

The electrochemical potential versus Na^0^/Na^+^ electrode of each step of sodiation is calculated and shown in Figure 8. As in the case of Li, two features can be outlined: the appearance of two high-voltage steps at 2.9 and 2.4 V at the beginning of the MOF sodiation, followed by a drastic decrease in the potential (by more than 1.0 V) for the last sodiation degrees. The high-voltage steps of MOF-sodiation are shifted in comparison with those corresponding to the MOF lithiation, which is in agreement with the standard redox potentials of sodium and lithium. This allows assigning the high-voltage steps to the preferential reduction of 2CN-BQ ligands by Na. However, the total amount of Na needed to reduce 2CN-BQ is lower than that for Li (i.e., 6 versus 8, Figure 5 and Figure 8). For the sake of comparison, the o-2CN-BQ grafted CNTs have been shown to exhibit lower capacity in a sodium half-cell than that in a lithium one [52]. The observed difference in the reduction of 2CN-BQ ligands by Na and Li is, most probably, associated with their coordination dissimilarity.

However, the MOF is still able to uptake extra Na due to the complementary redox properties of the Ni ion and CN nitrogens. The high degrees of sodiation take place at potentials lower than 1.2 V through several well-discernable steps. The total number of sodium uptake is 16 Na, which is lower than that of Li (i.e., 24). This difference arises from the specific feature of Li to form metal clusters in the reduced MOF. In comparison with Li, it appears that the formation of metal clusters is restricted for Na. This can be explained with the structural changes of more pronounced volume compactization.

#### 2.2.3. Magneziation of MOF-S21

**Figure 9:**
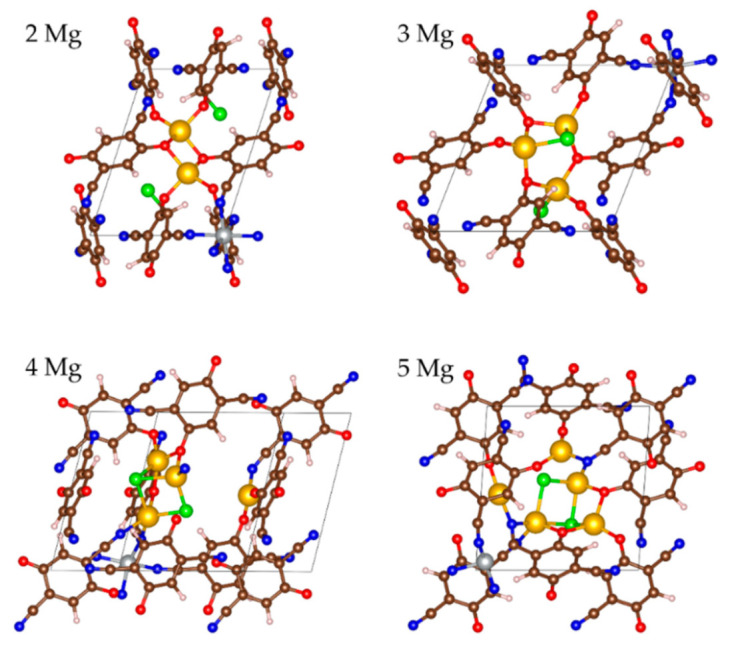
The MOF-S21 unit cell early stages of magnesiation. Color code: yellow—Mg, the remaining colors as in Figure 1.

As in the case of Li, Mg displays a selective coordination towards the functional groups and counterions in MOF-S21. The first two magnesium atoms are coordinated solely to C=O oxygens. Upon the introduction of the next two Mg atoms, first the chlorides and then the CN-nitrogens are engaged in the coordination (Figure 9). The maximum number of 6 Mg could be added energetically favorably.

**Figure 10:**
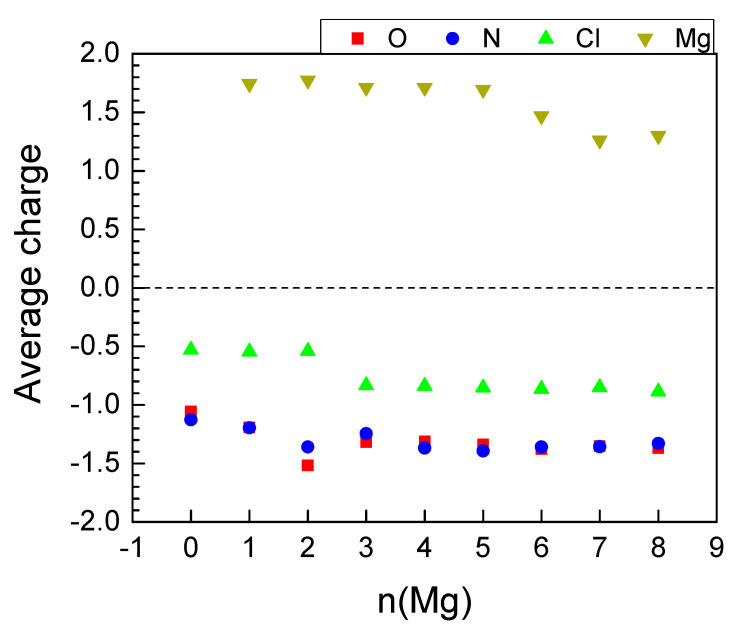
Average AIM charges upon magnesiation of MOF-S21.

**Figure 11:**
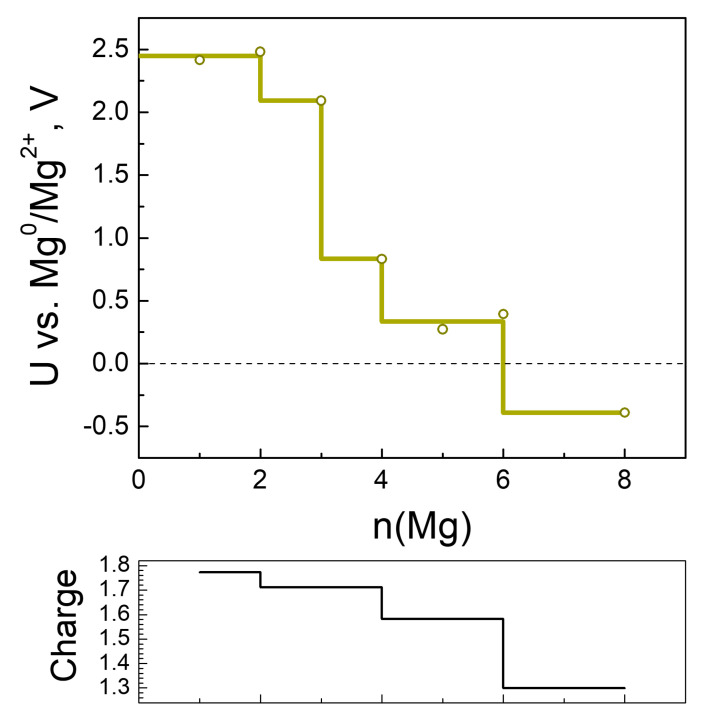
Calculated electrode potential profile (vs. Mg^0^/Mg^2+^) compared to Mg charge variation as a function of magnesiation.

The insertion of the first 4 Mg invoked practically no effect on the charges of Mg and Ni, nor on the coordination number of the latter (Table 4). The next two Mg atoms caused gradual drop in the charges of both metals and the coordination number of Ni, resulting in inefficient further magneziation (Table 4, Figure 10 and Figure 11—bottom). The BLA values reveal that the quinoid ring was aromatized as early as the insertion of 2 Mg and stayed aromatic, while BLA of the exocyclic fragment started decreasing at 4 Mg (Figure 4). Upon Mg insertion the volume of the unit cell underwent volume contraction, followed by a rapid expansion thereafter, most probably due to the partial Ni decoordination (Table 4). The expansion did not go beyond 95% of the initial unit cell volume. Moreover, the averaged periodic box parameters are the closest to those of the empty box, i.e., the unit cell withstands the least deformation upon Mg loading. No aggregation of Mg atoms is registered (Table 4).

As usual, the electron density from Mg went to oxygen and nitrogen; however, unlike Li and Na, the shares of O and N were equal (Figure 10) and kept essentially constant after 2 Mg. Therefore, at 6Mg and beyond, the Mg charge started decreasing and reduction of Ni was witnessed (Table 4, Figure 10).

The Mg charge variation profile is synchronized with the electrochemical potential profile, except upon the addition of 3 Mg (Figure 11). In comparison with Li and Na, the electrochemical curve of Mg consists of two high-potential steps at 2.4 and 2.0 V, followed by a large jump in the potential of over 1.5 V after 3 Mg. The high potentials corresponding to the uptake of 2 and 3 Mg are lower than those calculated for low degrees of MOF lithiation and sodiation, as could be expected from the magnitudes of the standard redox potentials of magnesium, sodium and lithium. This means that the high-voltage steps occurring during the MOF magnesiation are also a consequence of the preferential reduction of the 2CN-BQ ligands. Contrary to Li and Na, the MOF accommodates a limited number of extra Mg (i.e., only 3 Mg) at potentials lower than 1 V.

### 2.3. Generalized Electrochemical Results

Based on the results for the electrochemical potential, the theoretical specific capacity and energy density are calculated (Table 5). The highest capacity is delivered upon lithiation, while the lowest—upon magnesiation. The same order is obeyed for the energy density. To appraise these results, we referred to data for metal-organic batteries employing Li and Mg metal anodes, and anthraquinone as a cathode: the theoretical energy density is 530 W h/kg for the Li-cell and 345 W h/kg for the Mg-cell [53]. In addition, it has recently been found that a ferrocene-based MOF with composition iron (III) 1,1′-ferrocenedicarboxylate showed a high energy density of 549 W h/kg as a cathode in a lithium-ion battery [54]. The comparison manifests that the designed MOF exhibits twice as high energy density in respect to both Li^+^ and Mg^2+^. Moreover, the energy density of MOF-S21 towards Li^+^ exceeds that of Li-rich oxides, which are now considered as next-generation electrode materials for LIBs (i.e., energy density is higher than 400 W h/kg) [55].

In comparison with Li^+^ and Mg^2+^, the capacity and energy density upon MOF-S21 sodiation are intermediate. However, the magnitudes of both capacity and energy density outperform those predicted for the highest-performing layered oxides Na_2_RhO_3_ and Na_2_PdO_3_ [56]: energy density of 747 and 734 W h/kg, respectively.

**Figure 12:**
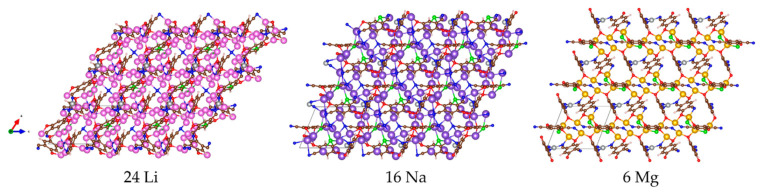
Multiple cell representation of the maximum exergonic uptake of MOF-S21 with the respective metal atoms.

The suitability of the designed MOF as an electrode material in Li, Na and Mg-ion batteries can also be evaluated by the chemical and geometrical stability upon metal insertion. MOF-S21 appears to be chemically stable since no covalent bond cleavage is observed at any level of metal uptake. The structural changes invoked by the maximum amount of favorably added Li, Na and Mg into MOF-S21 are compared on Figure 12. Li_24_-MOF exhibits the largest structural transformation: the unit cell deviates most significantly from the initial rhombohedral cell with a volume change of around 20%. In contrast, the unit cells of both Na_16_-MOF and Mg_6_-MOF are closer to the initial one with only 5 and 8% volume decrease, respectively. These are exceptionally small volume changes if we consider the ionic radius of Na^+^ versus that of Li^+^, as well as the charge of Mg^2+^ versus that of Li^+^. The limited volume changes will generate little stress and strain in the electrode during cycling, thus preventing its performance failure. Therefore, one can expect a better cycling stability of the MOF-S21 during sodiation and magnesiation in comparison with that of lithiation.

## 3. Models and Methods

All calculations were performed in periodic boundary conditions (PBC) with the Vienna Ab initio Simulation Package (VASP 5.4.4) [57,58,59]. Spin polarized density functional theory (DFT) was employed, specifically the PBE generalized gradient approximation exchange and correlation functionals [60]. The core electrons were described with pseudopotentials using the projector-augmented wave (PAW) method [61,62]; for the outer electrons, a plane-wave basis set with an energy cut-off of 600 eV was utilized. The used cores for the elements were as follows: [He] for Li, C, N and O; [Be] for Na and Mg; [Ne] for Cl; and [Ar] for Ni. The Γ-point was used to sample the Brillouin zone. The electronic partial occupancies were calculated according to the Gaussian smearing scheme with a smearing parameter of 0.05 eV. Geometry optimization, including the unit cell shape and volume, was carried out until all forces acting on the atoms became less than 0.01 eV/Å. The charge distribution was calculated according to Bader’s quantum theory of atoms in molecules (QTAIM) [63] with the Bader program [64]. The VESTA program [65] was used for structure visualization. The models with a small to medium amount of metal atoms were built by testing different chemically sensible interaction sites and proceeding with the most stable structure after optimization. Upon a certain degree of metalation, all intuitively chosen sites became occupied. In order to generate further initial structures, short molecular dynamics simulations were carried out. Computational details, unique to the MD simulations, included: Canonical ensemble; Nose-Hoover thermostat [66,67,68]; integration of the equations of motion according to the Verlet algorithm [69]; time step of 0.5 fs; temperature = 400 K; energy cut-off of 400 eV; 4000 or 6000 MD steps. The energies of bulk metals were calculated using the experimental cell parameters and an 11 × 11 × 11 k-point mesh.

The step-wise energy differences used for the estimation of the potential were calculated according to the equation:

(1) $$\Delta E_{\mathrm{DFT}}\;=\;E\left(M_{y}\mathrm{MOF}\right)-[E\left(M_{x}\mathrm{MOF}\right)+\left(y-x\right)E\left(M\right)]$$

where *x* and *y* are the number of metal atoms in two consecutive steps of metal insertion and E(M) is the energy of one atom in the bulk metal.

It has been shown that, for solid systems, the difference between the internal energy and the free energy is negligibly small and the electrode potential can be estimated using the equation [70,71]:

(2) $$U\;\left[V\right]=-\Delta E_{\mathrm{DFT}}\left[eV\right]/z$$

The capacity and the energy density of the electrode material are calculated according to the formulas:

(3) $$\mathrm{Capacity}=z*F/\left(3.6*m_{MOF}\right)$$

(4) $$\mathrm{Energy}\;\mathrm{density}=F/(3.6*m_{MOF})\int U\;dz$$

where *z* is the number of exchanged electrons, *F* is the Faraday constant, *m_MOF_* is the molar mass of the electrode material in g.mol^−1^.

## 4. Conclusions

Coordination of Ni(II) with CN-groups from 2,5-dicyano-*p*-quinone ligands leads to the formation of a rhombohedral MOF, the charge neutralization being achieved by Cl¯ counterions. The designed MOF is able to uptake an extremely high amount of alkali and alkaline earth metals, i.e., 24 Li, 16 Na and 6 Mg. The inserted metal atoms further lower the cell symmetry and convert it into a triclinic one, but in a different way—Li invokes maximum flattening and the smallest volume (about 20% volume drop), whereas Mg, and especially Na, have the opposite effect towards rectifying the angles (and no more than 8 and 5% volume decrease). This could be considered as a guarantee for a good cycling stability during the sodiation and magnesiation of the designed material. In all cases, the addition of metal atoms inflicts compactization of the MOF owed to structural changes in its edges—the quinone molecules. The latter gradually lose their quinoid character caused by carboxyl oxygen reduction and become aromatic. This is accompanied by strengthened delocalization in the exocyclic fragment due to CN reduction, leading to a subsequent requinoidization of the organic molecule in the N-N direction. The latter process results in partial (Na, Mg) or substantial (Li) reduction and decoordination of Ni(II). The charge redistribution and the electrochemical potential profiles confirm this description of the processes occurring upon introduction of Li, Na, and Mg. Each stage of involvement of MOF fragments in the reduction are reflected in the charge and potential profile of the respective added atom. The first quotas of added metals (i.e., 8 Li, 6 Na and 3 Mg) are stored in the MOF at two high-potential steps (i.e., 3.55/2.8 V vs. Li^0^/Li^+^, 2.9/2.4 V vs. Na^0^/Na^+^ and 2.4/2.0 V vs. Mg^0^/Mg^2+^) due to reduction of the 2CN-BQ ligands, while below 1.0 V, the MOF accommodated an extra number of metals through the activation of the redox properties of the Ni ion in addition to the CN-nitrogens. An additional feature of MOF-S21 is the overlithiation due to Li clusters formation.

The designed MOF stores Li^+^, Na^+^ and Mg^2+^ by delivering high specific capacity and energy density that significantly exceeds those typical for Li- and Na-rich layered oxides, as well as the organic anthraquinone-based electrodes. Moreover, MOF-S21 is definitely more environmentally friendly, as the contents of Ni is 9.7 wt%, while, in the conventional layered oxides, the transition metals (Ni, Co) constitute more than 50 wt%. Moreover, as reported recently [72], quinones could be extracted from biomass, which would make the production of quinone-based MOFs a green chemistry project. The predicted excellent electrochemical properties of MOF-S21 regarding Li^+^, Na^+^ and Mg^2^^+^ deserve to be tested experimentally.

## Figures and Tables

**Table 1:** AIM metal charges and some structural descriptors of the lithiated MOF-S21.

n(Li)	q_ave_(Li)	q(Ni)	Volume of Unit Cell, Å³	Coord. # of Ni R(CN…Ni) < 2.3 Å	# Li-Li Bonds R(Li-Li) < 2.67 Å
0	–	1.25	1024.8	6	
2	0.88	1.21	795.3	6	0
4	0.87	1.21	753.9	6	0
8	0.87	0.75	763.9	4	5
18	0.86	0.32	770.7	3	7
24	0.84	0.24	794.1	2	10
26	0.75	0.05	872.9	2	13

**Table 2:** Structural parameters of the periodic cell upon maximum exergonic metal insertion.

Metal	a, Å	b, Å	c, Å	α, °	β, °	γ, °
0	11.67	11.67	11.67	56.16	56.16	56.16
24 Li	10.79	11.77	12.08	50.27	43.85	54.06
16 Na	9.19	11.15	10.64	91.77	68.65	108.54
6 Mg	10.08	10.83	11.15	61.92	61.15	65.21

**Table 3:** AIM metal charges and some structural descriptors of the sodiated MOF-S21.

n(Na)	q_avr_(Na)	Volume of Unit Cell, Å³	q(Ni)	Coord. # of Ni R(CN…Ni) < 2.3 Å	# Na-Na Bonds R(Na-Na) < 3.08 Å
0	–	1024.8	1.25	6	
4	0.88	869.0	1.18	6	0
6	0.88	851.3	0.94	4	0
8	0.88	946.3	0.61	4	0
10	0.88	963.3	0.51	4	0
12	0.86	985.4	0.46	4	1
14	0.86	993.3	0.44	4	1
16	0.76	959.4	0.43	4	1
18	0.69	974.0	0.40	4	1

**Table 4:** AIM metal charges and some structural descriptors of the magnesiated MOF-S21.

n(Mg)	q_ave_(Mg)	Volume of Unit Cell, Å³	q(Ni)	Coord. # of Ni R(CN…Ni) < 2.3 Å	# Mg-Mg R(Mg-Mg) < 2.85 Å
0	-	1024.8	1.25	6	
2	1.77	944.4	1.24	6	0
3	1.71	802.8	1.24	6	0
4	1.71	770.6	0.82	5	0
5	1.69	750.1	0.79	4	0
6	1.47	910.9	0.74	4	0
8	1.30	950.6	0.14	2	0

**Table 5:** Electrochemical properties of MOF-S21 as an electrode material for different charge carriers. Capacity in mA h g^−1^ and specific energy in W h/kg.

Charge Carrier	Maximum Capacity	Gravimetric Energy Density
24 Li	1065	1343
16 Na	710	1025
6 Mg	533	753

## Data Availability

The input and output files from the calculations (including geometries and energies) are available from the corresponding author upon reasonable request.

## Supplementary Materials

The following are available online, Figure S1: MOF-S21 at different degrees of lithiation; Figure S2: MOF-S21 at different degrees of sodiation. Figure S3: Moderately lithiated and sodiated MOF-S21.
