# Supplementary material for: Redox Hyperactive MOF for Li+, Na+ and Mg2+ Storage
Source: Molecules. 2022 Jan 18;27(3):586. doi: 10.3390/molecules27030586 (PMC8838856; doi:10.3390/molecules27030586)
Supplement: Supplementary file 1 [file molecules-27-00586-s001.zip › molecules-1550099-supplementary.pdf]

Supplementary Materials for:

Redox Hyperactive MOF for Li^+, Na^+ and Mg^{2+} Storage

Hristo Rasheev,[1,2,*] Agnieszka Seremak,[1,3] Radostina Stoyanova [2] and Alia Tadjer [1,3]

[1] Faculty of Chemistry and Pharmacy, University of Sofia, 1164 Sofia, Bulgaria

[2] Institute of General and Inorganic Chemistry, Bulgarian Academy of Sciences, Sofia 1113, Bulgaria

[3] Faculty of Chemistry, Wroclaw University of Science and Technology, 50-373 Wroclaw, Poland

e-mail: fhhr@chem.uni-sofia.bg

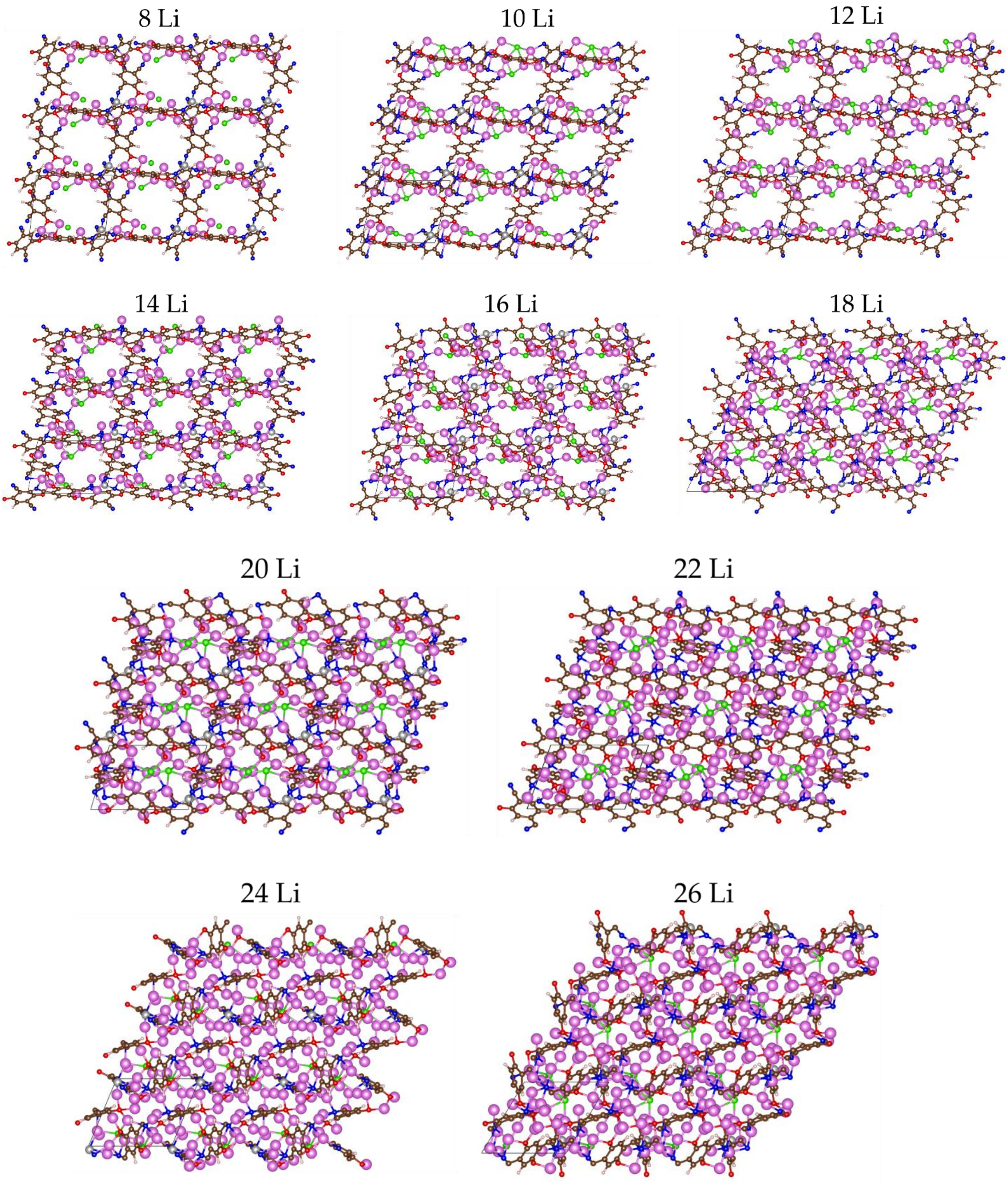

Figure S1. MOF-S21 at different degrees of lithiation

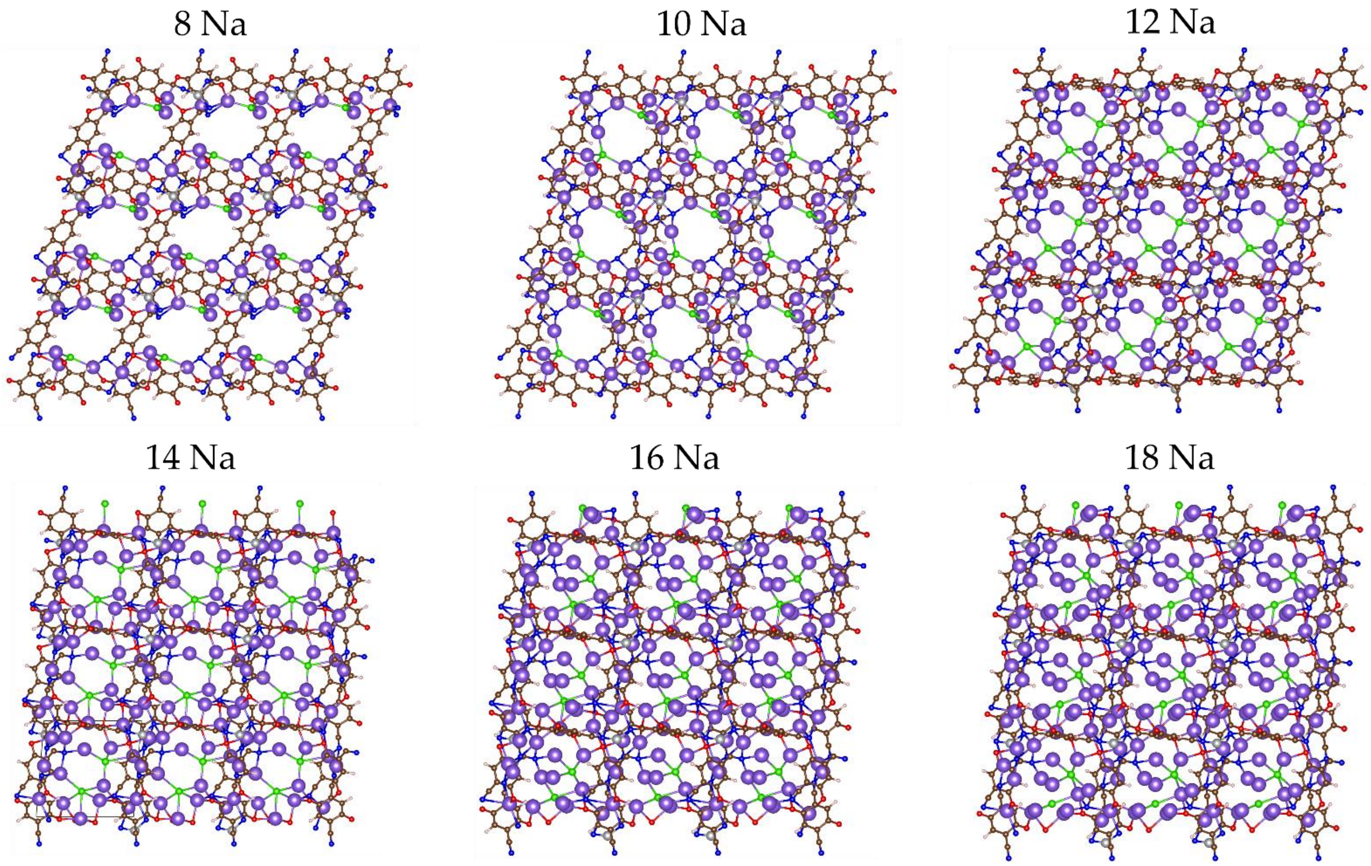

Figure S2. MOF-S21 at different degrees of sodiation

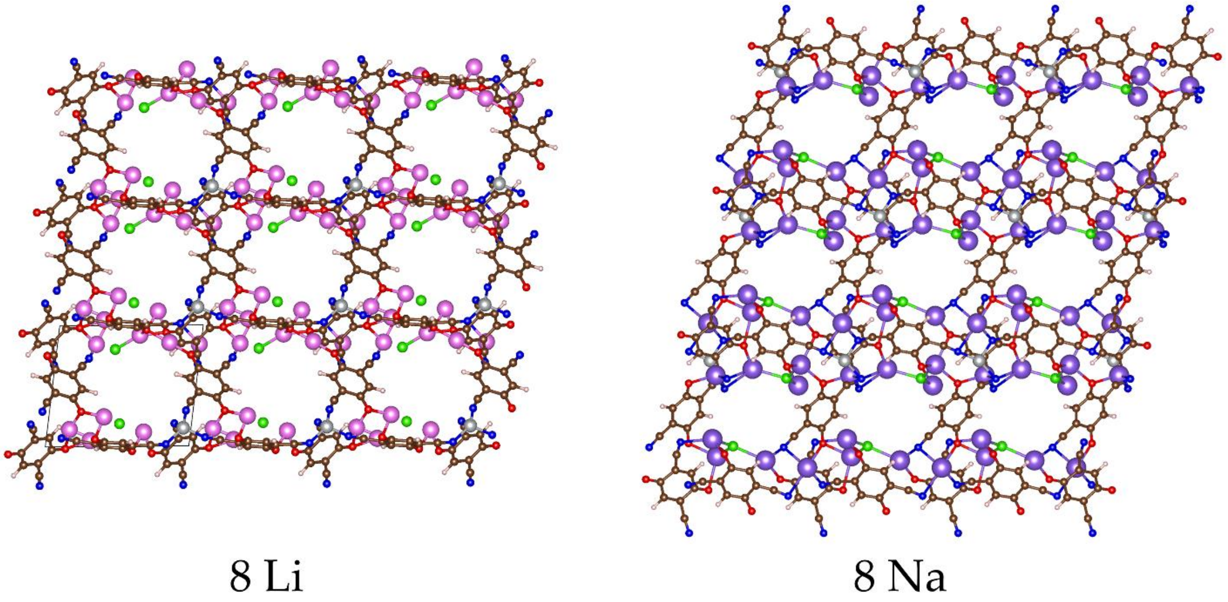

Figure S3. Upon intercalation of a moderate amount of Li and Na, the latter are situated close to the edges of the MOF, coordinated by electronegative centers such as O, N, Cl, so there is plenty of free space for migration. Even at this level of loading (M=8), one can spot some close contacts in the lithiated MOF, which are missing in the sodiated one.
